# FPCR-Net: Front Point Cloud Regression Network for End-to-End SMPL Parameter Estimation

**DOI:** 10.3390/s25154808

**Published:** 2025-08-05

**Authors:** Xihang Li, Xianguo Cheng, Fang Chen, Furui Shi, Ming Li

**Affiliations:** 1 College of Mechanical and Automotive Engineering, Ningbo University of Technology, Ningbo 315336, China; chenfang@nbut.edu.cn (F.C.); shifurui@nbut.edu.cn (F.S.); 2 Fenghua Research Institute of Ningbo University of Technology, Ningbo 315211, China; 3 Shanghai Key Laboratory of Intelligent Manufacturing and Robotics, Shanghai University, Shanghai 201900, China; helloming@shu.edu.cn

**Keywords:** parametric body modeling, parameter regression, front body scan, supervised learning

## Abstract

Due to the challenges in obtaining full-body point clouds and the time-consuming registration of parametric body models, we propose an end-to-end Front Point Cloud Parametric Body Regression Network (FPCR-Net). This network directly regresses the pose and shape parameters of a parametric body model from a single front point cloud of the human body. The network first predicts the label probabilities of corresponding body parts and the back point cloud from the input front point cloud. Then, it extracts equivariant features from both the front and predicted back point clouds, which are concatenated into global point cloud equivariant features. For pose prediction, part-level equivariant feature aggregation is performed using the predicted part label probabilities, and the rotations of each joint in the parametric body model are predicted via a self-attention layer. Shape prediction is achieved by applying mean pooling to part-invariant features and estimating the shape parameters using a self-attention mechanism. Experimental results, both qualitative and quantitative, demonstrate that our method achieves comparable accuracy in reconstructing body models from front point clouds when compared to implicit representation-based methods. Moreover, compared to previous regression-based methods, vertex and joint position errors are reduced by 43.2% and 45.0%, respectively, relative to the baseline.

## 1. Introduction

In recent years, with the development of fields such as virtual reality and virtual fitting, human body reconstruction technology based on 2D images or 3D point clouds has garnered significant attention [1,2]. Among them, 2D images, due to their convenient acquisition, became the focus of early research. However, due to the lack of depth information, methods of human body reconstruction based on 2D images have inherent limitations in terms of the accuracy of body shape and pose reconstruction [3]. In contrast, 3D point clouds retain the geometric structure of the human body, making them more favored in high-precision application scenarios such as sports medicine analysis, virtual fitting, and immersive entertainment. Traditionally, high-precision full-body 3D scanning systems can acquire complete body point clouds, which are ideal inputs for parametric reconstruction [4]. However, due to their large size and high cost, their application is limited [5]. With the popularity of consumer-grade depth cameras (such as Kinect [6]), 3D reconstruction technology based on single-viewpoint point clouds has developed rapidly. Among them, front point clouds, which contain more information about key human body parts (such as facial, chest, and limb contours) and are easy and natural to acquire, have become the most commonly used input form. Existing reconstruction methods usually adopt a two-step strategy: first predicting full-body shape, and then registering the standard model (such as SMPL [7]) through the Iterative Closest Point (ICP) [8] algorithm or its variants [4,9]. However, the iterative optimization registration process based on the ICP algorithm has high computational cost and slow processing speed, making it difficult to meet real-time requirements for dynamic human body modeling and interactive applications.

Some researches attempt to directly regress a parametric body model from point clouds, aiming to eliminate the registration step, but the results are often unsatisfactory [10,11]. This is primarily because the body shape information (such as height and curves) in point clouds is highly coupled with pose information (such as limb angles and movements), making it challenging for regression networks to simultaneously distinguish and learn these two types of features [10]. When different body parts are too close together in point clouds, their features may become mixed during convolution operations, further interfering with the model’s predictive ability. Additionally, the limitations of single-view frontal point cloud input also affect model performance. Many body parts, especially the shape information of the back and sides, may be partially or completely missing, making it difficult for the model to comprehensively reconstruct and predict the 3D shape of the human body.

To address these challenges, we propose FPCR-Net (Front Point Cloud Regression Network), a novel end-to-end SMPL parameter regression network tailored for single-view front point clouds. The design of FPCR-Net is motivated by the need to overcome the limitations of missing geometric information and feature interference in single-view data. Our approach introduces a back point cloud prediction module to infer missing back geometry, leveraging the approximately cylindrical nature of human body parts to establish point correspondences within the same body part. Additionally, FPCR-Net extracts SO(3) equivariant features separately from front and predicted back point clouds using a lightweight PointNet++ [12] with separable discrete convolutions (SPConv) [13], mitigating rotational ambiguity and interference from non-articulated parts in close proximity. These features are aggregated into part-equivariant and part-invariant features for pose and shape regression, respectively, using soft aggregation and self-attention mechanisms to ensure robust and differentiable predictions. Experimental results show that FPCR-Net significantly improves accuracy over baseline regression methods and matches implicit representation methods with much faster reconstruction. However, self-occlusion in complex poses can cause information loss, so our method targets relatively complete front point cloud inputs. The main contributions of this paper can be summarized as follows:

1. FPCR-Net is the first network designed to directly regress SMPL pose and shape parameters from single-view front point clouds, eliminating the need for iterative registration.
2. FPCR-Net extracts SO(3) equivariant features separately from front and back point clouds to reduce rotational ambiguity and interference, while employing soft aggregation and self-attention mechanisms for differentiable, accurate SMPL pose and shape regression.

## 2. Related Work

### 2.1. Advances in Human Body Reconstruction

Human body reconstruction has made significant progress due to its applications in virtual reality, motion capture, and virtual fitting. Early methods relied on 2D images, using contour, edge, or keypoint information to estimate 3D body shapes and poses [1,14]. However, these approaches were limited by depth ambiguity, restricting their accuracy in capturing detailed geometries. To address this issue, parametric models such as SMPL [7] and SMPL-X [15] have been widely adopted, as they provide low-dimensional representations of body shape and pose, enabling robust reconstruction from various inputs. For instance, SHAPY [16] significantly improves 3D shape estimation from images by utilizing body measurements and shape attributes. SMPLify-XMC [17] generates highly realistic shapes and poses by optimizing SMPL-X parameters, leveraging contact constraints and known 3D poses. PostureHMR [18] estimates 3D human poses from 2D images through a multi-step diffusion process and a kinematics-based forward process, and it introduces an M2P decoder and trimap-based rendering loss to effectively improve pose estimation accuracy. However, the inherent ambiguities in the 2D to 3D transformation limit the applicability of these methods for high-precision predictions.

To mitigate spatial ambiguities in body reconstruction, methods for deriving parametric body models from point cloud have also garnered significant attention. Currently, the majority of body reconstruction methods based on input point clouds predominantly employ a two-stage strategy: initially, they regress intermediate representations of parametric body models (such as reference point clouds [19], skeletal joint locations [20], corresponding body parts [21,22], etc.); subsequently, these representations are utilized as initialization for fine-grained registration optimization, thereby achieving accurate and robust reconstruction of parametric body models. This approach demonstrates superior performance when processing sparse and noisy point cloud inputs. Although directly regressing the parameters of parametric model from point cloud is highly attractive, the inherent unstructured nature of point cloud and the high degree of coupling between body shape and pose present numerous challenges for directly regressing SMPL parameters [10]. The only known work that can regress SMPL parameters directly from point clouds is ArtEq [11], which learns a part detection network by leveraging local SO(3) invariance, and regress shape and pose using articulated SE(3) shape-invariant and pose-equivariant networks. However, ArtEq’s design is targeted at full-body complete point cloud. When the input is a single-view point cloud, this method faces the challenge of dealing with almost all body parts with partially missing or completely missing shapes. Directly regressing SMPL parameters from such incomplete point cloud data can lead to severe instability. This paper continues the core concept of separating the shape and pose of body point cloud in ArtEq, and proposes a new method aimed at overcoming the challenges posed by single-view point cloud input, while maintaining the direct regression capability for SMPL parameters.

### 2.2. Advances in Point Cloud Completion

Point cloud completion aims to reconstruct complete 3D shapes from incomplete inputs, such as front-view point clouds, which is particularly important for human body reconstruction tasks, as single-view point clouds often lack information about the back or sides due to occlusion, viewpoint limitations, or low-resolution sensors. Early methods relied on geometric assumptions, such as symmetry or template fitting, but performed poorly on complex poses and discontinuous surfaces, such as body joint areas [23]. The rise of deep learning has significantly enhanced the robustness of point cloud completion, with PointNet [24] and PointNet++ [12] laying the foundation for handling unordered point sets.

Recent research on point cloud completion has made significant progress. Transformer-based methods, such as PoinTr [25] and AdaPoinTr [26], achieve high-quality completion by learning global shape priors, but their performance is limited when handling non-uniformly sparse point clouds in real-world scenarios. ProxyFormer [27] uses point proxies and a missing-part sensitive transformer to effectively predict missing points, achieving good performance and fast reasoning speed; PCoT-Net [28] leverages a point contextual transformer and an External Attention enhanced feature completion module to effectively capture fine-grained local contextual features, outperforming existing methods in both quantitative performance and visual quality. Attention-based mechanisms such as PointAttN [29] leverages cross-attention and self-attention mechanisms to implicitly partition local regions, capturing structural relationships among points and enhancing the prediction of complete 3D shapes with detailed geometry.

Although the aforementioned methods perform excellently in general point cloud completion, they are typically designed for common objects and struggle to fully address the specific characteristics of human body point clouds, such as self-occlusion or interference when body parts are in close proximity. The method proposed in this paper introduces a back point cloud prediction module based on human body parts, employing a cylindrical approximation strategy to mitigate self-occlusion, and improves feature extraction to support SMPL parameter regression, thereby filling the gap in existing methods for human body point cloud completion.

## 3. Method

**Figure 1:**
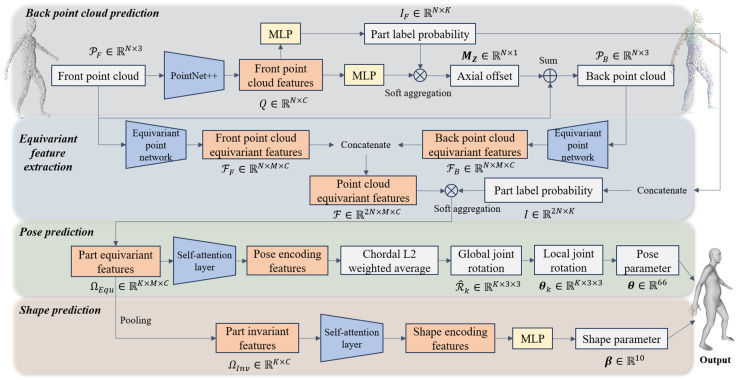
Architecture of FPCR-Net for SMPL shape and pose parameter regression from a body front point cloud.

Given an input of a body front point cloud $P_{F}\in R^{N\times 3}$ (where *N* denotes the number of points), FPCR-Net aims to regress the SMPL shape parameter $\beta \in R^{10}$ and pose parameters $\theta \in R^{66}$. FPCR-Net first utilizes the PointNet++ network to extract features from the input point cloud and predict the corresponding back point cloud as well as the probability of each point belonging to a body part. Subsequently, FPCR-Net designs an equivariant point network to extract equivariant features of the front and back point clouds separately, and then performs weighted aggregation on these features based on the part label probability to obtain part-level equivariant features. To effectively process these features and maintain equivariance, FPCR-Net introduces a self-attention mechanism. In the pose parameter estimation stage, FPCR-Net transforms the pose regression problem into a weight prediction problem, obtaining the final pose by weighted averaging the rotation elements. Finally, FPCR-Net pools the part-level equivariant features on the rotation group to obtain part-invariant features and predicts body shape parameters based on these features. The entire network consists of four modules, responsible for back point cloud prediction, equivariant feature extraction, pose prediction, and shape prediction, respectively. Its structure is shown in Figure 1. Detailed descriptions of each module design are as follows.

### 3.1. Back Point Cloud Prediction

**Figure 2:**
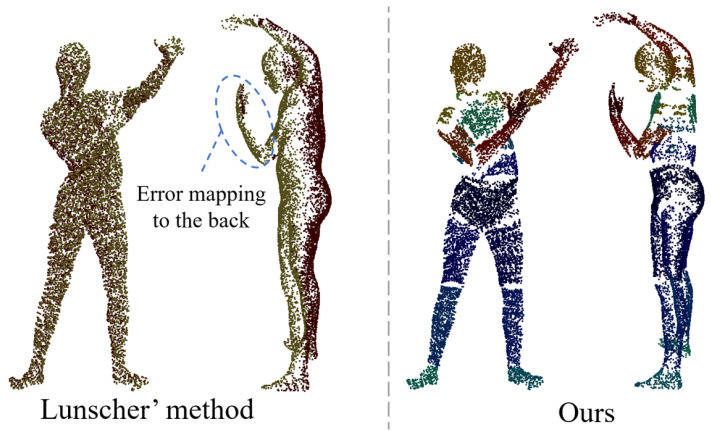
Comparison of back point cloud prediction methods.

Since we only use a front point cloud as input, the lack of a back point cloud leads to a large uncertainty in the description of the body geometry, especially in terms of body shape perception. In addition, there is also ambiguity about the rotation of the joints in certain self-obscuring cases, such as the difficulty of determining the rotation of the forearm from a single-view point cloud alone in the case of an occluded hand. Therefore, predicting the back point cloud is necessary. Lunscher et al. [23] extracted the smallest self-occluded surface containing the body, via a depth map-fixed axis, to obtain a more complete point cloud. However, this method only considers the A-pose case and fails to deal with the interference when there is occlusion of different body parts. For example, when the hand is bent over the chest, the hand point cloud is incorrectly mapped to the back, which introduces huge uncertainty and makes it difficult for the network model to learn, as shown on the left of Figure 2.

**Figure 3:**
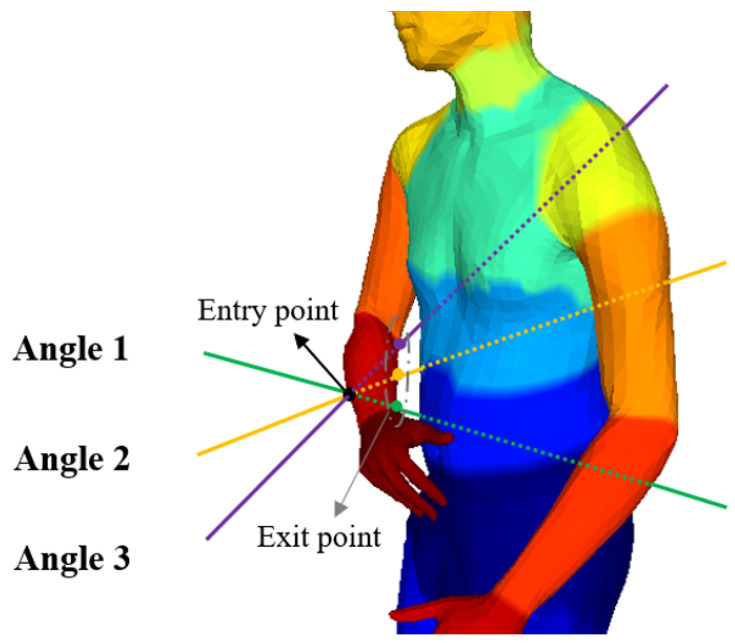
Schematic diagram of ray entry and exit of the same body parts. Different colors represent different body parts.

In order to overcome the ambiguity caused by Lunscher et al.’s method of predicting the corresponding points of the back point cloud directly from the front point cloud, we base our approach on the fact that most parts of the human body (e.g., torso, legs, and arms) are approximately cylindrical. When a ray enters from a point on the side of the cylinder, the exit point on the cylinder can be found in most cases, and the intersection is usually unique (there may be multiple points of intersection in the finger region, and the point furthest from the entry point is used as the exit point), as shown in Figure 3. Therefore, it is feasible to learn the correspondence between the front point cloud and the back point cloud within the same body part (in this paper, the body parts are divided into 22). For the front point cloud, it is assumed that the camera plane is perpendicular to the *Z*-axis, so only the offset of the front point cloud on the *Z*-axis needs to be learned to compute the corresponding back point cloud, as shown on the right of Figure 2.

To constrain the prediction of the back point cloud within the same body part, we also predict body part labels. However, hard classification based on argmax operations is non-differentiable, and it becomes challenging to find corresponding back points at part boundaries or when the angle between the ray and cylindrical surface is large. Therefore, we first extract the point cloud feature representation $Q\in R^{N\times C}$ (*C* = 64 is the feature dimension) using PointNet++ [12], followed by decoding it via MLP to output the part label probability $I_{F}\in R^{N\times K}$ (*K* = 22 is the number of part labels). Finally, the axial offset features generated by the MLP decoding are weighted to output the axial offset $M_{z}$ using the part label probability $I_{F}$ through a soft aggregation mechanism:

(1) $$M_{z}=\sum_{i}^{N}I_{F}\left(x_{i},p_{k}\right)L\left(x_{i}\right)$$

where $M_{z}\in R^{N\times 1}$ represents the *Z*-coordinate offset matrix corresponding to the input $P_{F}$, $L\left(x_{i}\right)$ denotes the axial offset feature of the point $x_{i}\in P_{F}$, and $I_{F}\left(x_{i},p_{k}\right)$ denotes the probability that the point $x_{i}$ belongs to the body part $p_{k}$.

For the back point cloud $P_{B}\in R^{N\times 3}$, it is only necessary to sum $M_{z}$ with the input $P_{F}$ on the *Z*-axis:

(2) $$P_{B}=P_{F}+\left[\begin{array}{c} 0\\ 0\\ M_{z} \end{array}\right]$$

### 3.2. Point Cloud Equivariant Feature Extraction

**Figure 4:**
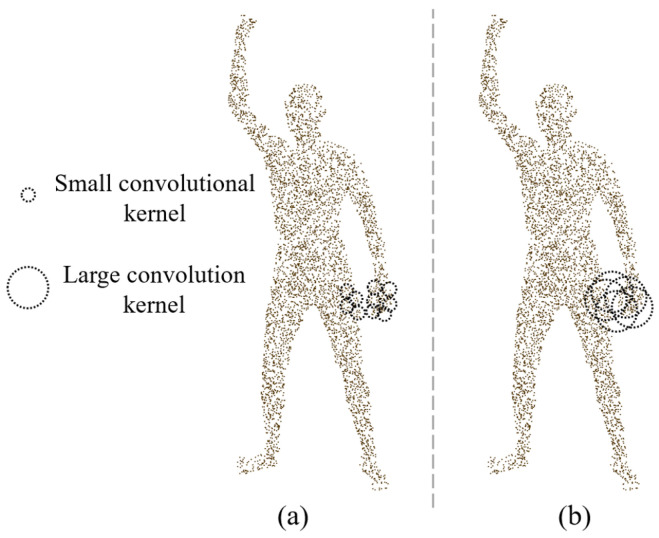
Schematic diagram of extracting local SO(3) equivariant features. (**a**) Using small convolution kernel. (**b**) Using large convolution kernel.

It is noteworthy that equivariant point networks exhibit poor feature extraction capabilities for rotationally symmetric shapes [13], and many parts of the human body are approximately cylindrical. The concatenation process of front and back point clouds may lead to rotational ambiguity in the extracted equivariant features during subsequent feature aggregation at the part level. In addition, the human body is a multi-part articulated structure and equivariant feature extraction may fail when the non-articulated parts are in close proximity to each other. This is because the convolution kernel may contain points where the geodesic is far away (for example, when the hand is close to the thigh, the geodesic distance may be farther away, but these points are geometrically closer in Euclidean space, as shown in Figure 4). This phenomenon may lead to incorrect aggregation of equivariant features, affecting the accuracy of feature representation.

**Figure 5:**
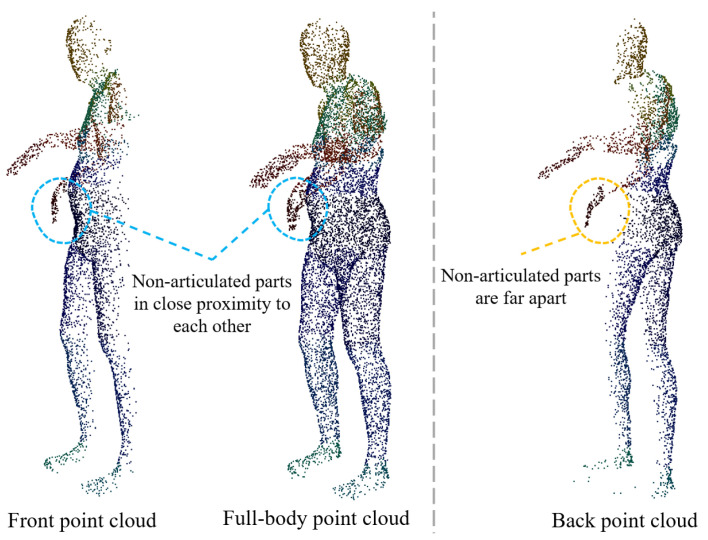
Schematic diagram of the reasons for separate extraction of SO(3) equivariant features for front and back point clouds.

In order to alleviate the above problems, we adopt the scheme of extracting SO(3) equivariant features of front and back point clouds, respectively. The theoretical basis of this method is that the geometric distribution of the local region is more unitary in the front and back point clouds, avoiding the interference of rotation ambiguity in the full-body point cloud. In addition, it can alleviate the influence of equivariant feature extraction when non-articulated parts are close to each other. Taking the pose shown in Figure 5 as an example, in the front point cloud and the full-body point cloud, the right hand is very close to the abdomen, and this spatial proximity will interfere with the SO(3) equivariant feature extraction of the point cloud. However, in the predicted back point cloud, the distance between the right hand and other non-articulated body parts is far, so the interference is less. At the same time, the SPConv layer of the equivariant point network uses a smaller convolution kernel size and is reduced to two layers to limit the receptive field of the network in a smaller area, so as to further reduce the interference caused by the proximity of non-articulated parts (Figure 4).

For specific implementation, taking the front point cloud as an example, with $P_{F}$ and the rotation group G of order |G|=M as input (where *M* = 60, as the icosahedron has 60 rotational symmetries), the model returns the SO(3) equivariant feature $F_{F}\in R^{N\times M\times C}$, representing the *C* dimensional feature vectors for each point in $P_{F}$ across the *M* group elements. Similarly, the back point cloud SO(3) equivariant feature $F_{B}\in R^{N\times M\times C}$ is obtained. Finally, the equivariant features of both the front and back point clouds are concatenated to form the global point cloud equivariant feature $F=\left\{F_{F},F_{B}\right\}\in R^{2N\times M\times C}$.

### 3.3. Pose Parameter Prediction Module

The point cloud equivariant feature F is defined at the level of points, while the SMPL pose parameters are defined on the body parts connected by joints. Therefore, it is necessary to aggregate F into part-based equivariant features to predict pose parameters. Since the back point cloud is predicted within the same part, the front and back point clouds share the same part label probability for a set of corresponding points; so, the part label probability corresponding to F is $I=\left\{I_{F},I_{F}\right\}\in R^{2N\times K}$ even though the features within the same part label can be averaged to obtain unique equivariant features with a size of M×C for each part. However, hard classification based on argmax is not differentiable, and the transition between adjacent body parts is blurred. Here, soft aggregation is also used to perform weighted averaging on the point cloud equivariant features according to the part label probability *I*:

(3) $$\Omega_{Equ}\left(p_{k},g_{j}\right)=\sum_{i}^{2N}I\left(x_{i},p_{k}\right)F\left(x_{i},g_{j}\right)$$

where $\Omega_{Equ}\in R^{K\times M\times C}$ is the part-equivariant features, $I\left(x_{i},p_{k}\right)$ denotes the probability that the point $x_{i}$ belongs to the body part $p_{k}$, and $F\left(x_{i},g_{j}\right)$ denotes the equivariant feature of the point $x_{i}$.

It is unreliable to directly estimate the pose parameters of each joint in SMPL, as its joint rotations are defined relative to the parent joint in the kinematic tree, while the equivariant features are defined in the global coordinate system. Therefore, we estimate the global rotation matrix ${\hat{R}}_{k}\in R^{K\times 3\times 3}$ for each part, which represents the rigid transformation of the part relative to the standard pose under the current pose. The local rotation matrix $\theta_{k}\in R^{K\times 3\times 3}$ can be calculated using the following equation:

(4) $$\theta_{k}={\hat{R}}_{k}\cdot \theta_{\mathrm{parent}\;(k)}^{T}$$

where $\theta_{\mathrm{parent}\;(k)}\in R^{K\times 3\times 3}$ is the accumulated rotation matrix of the parent joint.

Furthermore, to predict the part rotation matrix ${\hat{R}}_{k}$, a function is required to transform the part feature vector $\Omega_{Equ}$ into a suitable representation of ${\hat{R}}_{k}$. This function is constructed to maintain equivariance. Considering the computational complexity of SPConv layers, and leveraging the fact that rotational equivariance in continuous space is equivalent to permutation equivariance in discrete space, the part feature vector $\Omega_{Equ}$ is processed in discrete space. Since self-attention mechanisms possess permutation equivariance, self-attention on group elements can be employed to efficiently extract relationships between G elements, resulting in pose encoding features while maintaining equivariance.

The prediction of the global rotation matrix ${\hat{R}}_{k}$ can be regarded as a weighted aggregation of the probabilities of the group elements’ rotations. Here, the part-based group element feature $\Omega_{Equ}\left(p_{k},g_{j}\right)$ is used to regress the weights of each of the *M*-group elements. The final computed ${\hat{R}}_{k}$ is the Chordal L2 mean [30] of the group elements with the predicted weights.

Finally, the global rotation matrix ${\hat{R}}_{k}$ for each body part is converted to the local rotation matrix $\theta_{k}$ based on the SMPL kinematic tree using Equation (4). This is further transformed into the original axis-angle representation, thus achieving the estimation of SMPL pose parameters θ.

### 3.4. Shape Parameter Prediction Module

To robustly estimate body shape under varying observation conditions, it is necessary to ensure that the features remain invariant to these conditions. Therefore, $\Omega_{Equ}$ undergoes mean pooling across the group element dimension *M*, resulting in part-invariant features $\Omega_{Inv}\in R^{K\times C}$. This feature is further processed by a self-attention layer to capture the correlations between different body parts. After processing by the self-attention layer, the feature matrix is fed into an MLP to produce the final β parameters.

### 3.5. FPCR-Net Construction Details

In the back point cloud prediction, the part label probability prediction is performed using a hidden layer with feature dimensions of [B,64,N], where *B* is the batch size. The output is a part label probability matrix with dimensions [B,N,22]. The *Z*-coordinate offset prediction is conducted through a hidden layer with feature dimensions of [B,64×22,N], and the output is the point cloud offset features of size [B,22,N]. A reshape operation is then applied to convert this into the form [B,1,22,N], followed by a weighted summation with the Softmax probabilities of the part labels using Equation (1), resulting in the *Z*-coordinate offset matrix.

The equivariant feature extraction network consists of two SPConv layers, each with a kernel size of 0.4 and a stride downsampling factor of 2, designed to extract local SO(3) features from the point cloud.

In the pose prediction module, the self-attention layer utilizes a 2-dimensional embedding space, a 128-dimensional value space, 8 attention heads, and 2 stacked layers. Additionally, a linear-layer ReLU is attached to compute the Chordal L2 mean weights, since the self-attention layer does not include non-linear activations. The final output is the pose encoding features of size [B×22,60,128].

In the shape prediction module, the self-attention layer also uses a 2-dimensional embedding space, but with a 6-dimensional value space, 8 attention heads, and 2 stacked layers, outputting shape encoding features of size [B×22,6]. Finally, an MLP with a hidden layer of size [B,10] produces the shape parameters with an output size of [B,10].

## 4. Dataset Construction

In this work, the parametric body regression task from the input front point cloud is performed on the open-source CAPE dataset [31]. The point cloud equivariant features are aggregated into part-equivariant features through part label probabilities, which are then used to compute joint rotations. Therefore, the body should be segmented into parts according to the SMPL kinematic tree joints. To compute vertex labels, the SMPL model’s linear blend skinning weight matrix W is utilized, where $W\in R^{6890\times 24}$. This matrix represents the weight relationships between the 6890 vertices of the SMPL model and its 24 skeletal joints. For each vertex’s part label, the index of the maximum value in the corresponding weight vector is selected to determine the label:

(5) $$\mathrm{label}\left(v_{i}\right)=\underset{j\in \{1,\ldots ,24\}}{\mathrm{argmax}}W_{ij},\forall i\in \left\{1,2,\ldots ,6890\right\}$$

where $v_{i}$ represents the *i*-th vertex, and $W_{ij}$ denotes the weight in the *i*-th row and *j*-th column of the weight matrix W.

In the implementation, fingers are merged into the hands, and toes are merged into the feet, resulting in a segmentation of *K* = 22 body parts. In fact, we also disregard the prediction of pose parameters for these joints. Using the SMPL model parameters from the CAPE dataset (ignoring the pose parameters for fingers and toes), the corresponding SMPL model is generated. Then, the OpenDR [32] depth rendering engine is employed to randomly render depth maps within a range of −10° to +10° for both horizontal and pitch angles. The depth value of each pixel is then mapped to its corresponding coordinate in 3D space, generating a front point cloud $P_{F}$. In order to simulate the point cloud noise in the real environment, the front point cloud $P_{F}$ is processed with data enhancement. Specifically, Gaussian distributed noise with a mean of 0 and a standard deviation σ is randomly added to each point coordinate of the front point cloud, where the standard deviation σ is used to control the noise intensity. In the experiment, we randomly select the noise intensity as σ={0,0.01,0.05,0.1} to be added.

Subsequently, through nearest neighbor query algorithm, the part labels of the SMPL model vertices are transferred to each rendered point p.

(6) $$O\left(p\right)=\underset{z\in M}{\mathrm{argmin}}∥p-z∥$$

where $O\left(p\right)\in R^{22}$ denotes the part label of point p, M denotes the SMPL model mesh surface, and z is the M vertex.

For each point p, a ray is cast along the *Z*-axis towards the body to find the intersection points with the SMPL mesh. The set of intersection points is denoted as ℓ(p). Then, for each intersection point q∈ℓ(p), the nearest-point search algorithm is used to determine its part label O(q):

(7) $$O\left(q\right)=\underset{z\in M}{\mathrm{argmin}}∥q-z∥$$

Under the condition that the part labels are the same, the farthest intersection point from p is selected (since some areas, like the fingers, may have multiple intersection points) as the back point B(p).

(8) $$B\left(p\right)=\underset{q\in \ell (p),O(q)=O(p)}{\mathrm{argmax}}∥p-q∥$$

The back point cloud $P_{B}$ is the set consisting of all back points corresponding to the front points.

(9) $$P_{B}=\left\{B\left(p\right)|p\in P_{F}\right\}$$

**Figure 6:**
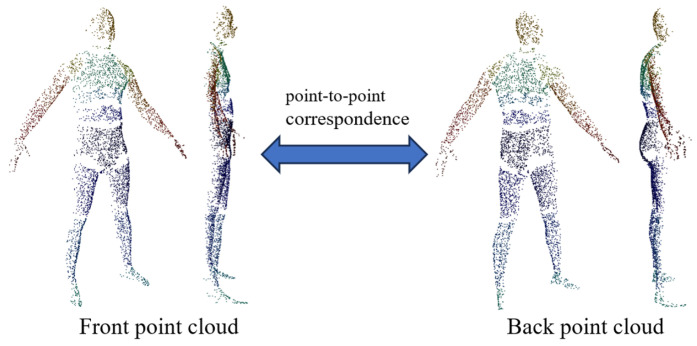
The corresponding front and back point clouds in the dataset.

Due to the constraint of limiting corresponding point searches to within the same part label, not all front points can find corresponding back points. In such cases, these points are removed to ensure strict correspondence in the training data. The corresponding front and back point clouds are illustrated in Figure 6. It is important to note that the purpose of the back point cloud prediction network is to learn the correspondence between front and back points. The part label probabilities are incorporated into the inference process through soft aggregation, enabling the network to still process complete front point clouds as input during inference.

## 5. Training Details

**Figure 7:**
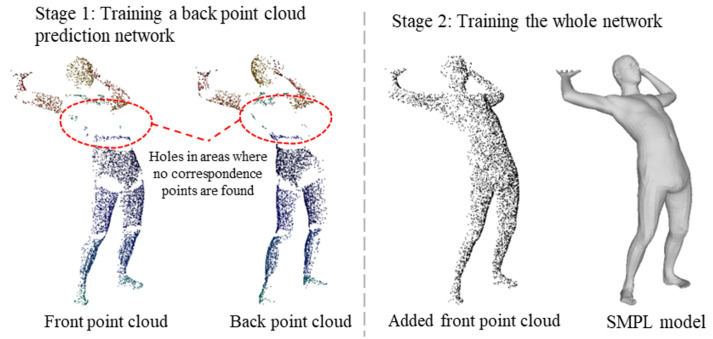
Schematic diagram of supervision data at each stage.

As previously mentioned, the search for corresponding points in the front and back point clouds is limited to the same part label. However, the body is divided into 22 parts, which in some poses leads to many front points being unable to find their corresponding back points. To maintain strict correspondence in the training data, the removal of these front points results in a large number of holes (as illustrated in the left image of Figure 7). Although this does not affect the back point cloud prediction network training and inference, the equivariant point network is sensitive to the point cloud distribution during point cloud SO(3) equivariant feature extraction. Large holes can affect point cloud equivariant feature extraction, and this is not consistent with the actual input situation. Therefore, we divide the training process into two stages. In the second stage, we introduce a complete front point cloud, so that the training data contain both the cases with holes and the complete samples of the point cloud. In this way, the diversity of training data is ensured, so as to improve the generalization ability of the model in processing various point cloud data. The supervisory data for each stage are shown in Figure 7, and the training process of each stage is explained in detail as follows.

In Stage 1, the back point cloud prediction network is trained. Taking the front point cloud with correspondence in our dataset as input, the loss function includes the following.

The cross-entropy loss $L_{\mathrm{part}\;}$ between the predicted body part label $I_{F}\left(x_{i},p_{k}\right)$ and the ground truth body part label $\tilde{I_{F}}\left(x_{i},p_{k}\right)$ of the point cloud:

(10) $$L_{\mathrm{part}\;}=\;\mathrm{cross}-\mathrm{entropy}\;\left(I_{F}\left(x_{i},p_{k}\right),\tilde{I_{F}}\left(x_{i},p_{k}\right)\right)$$

The Mean-Squared Error (MSE) loss $L_{\mathrm{back}\;}$ between the predicted back point cloud $P_{B}$ and the ground truth back point cloud $\tilde{P_{B}}$:

(11) $$L_{\mathrm{back}\;}={∥\tilde{P_{B}}-P_{B}∥}^{2}$$

Therefore, the loss function for this stage of the training process is

(12) $$L_{\mathrm{stage}\;1}=\lambda_{\mathrm{part}\;}L_{\mathrm{part}\;}+\lambda_{\mathrm{back}\;}L_{\mathrm{back}\;}$$

where $\lambda_{\mathrm{part}\;}$ = 1 and $\lambda_{\mathrm{back}\;}$ = 100.

In Stage 2, the parameters of the back point cloud prediction network are fixed and the complete front point cloud is used as input to train the entire SMPL parametric regression network end-to-end. The loss function is included as follows.

The MSE loss $L_{\mathrm{pose}\;}$ between the predicted pose parameter θ and the ground truth pose parameter $\tilde{\theta }$:

(13) $$L_{\mathrm{pose}\;}={∥\tilde{\theta }-\theta ∥}^{2}$$

The MSE loss $L_{\mathrm{shape}\;}$ between the predicted shape parameter β and the ground truth shape parameter $\tilde{\beta }$:

(14) $$L_{\mathrm{shape}\;}={∥\tilde{\beta }-\beta ∥}^{2}$$

The reconstructed SMPL mesh vertices v are compared to the ground truth vertices $\tilde{v}$ the weighted MSE loss $L_{\mathrm{verts}\;}$. ω is the weight of each vertex, where the weight of the labeled vertices is set to 2 and the weight of the remaining vertices is set to 1.

(15) $$L_{\mathrm{verts}\;}={∥\omega \left(\tilde{v}-v\right)∥}^{2}$$

The MSE loss $L_{joint}$ between the predicted SMPL mesh joint coordinates *J* and the ground truth joint $\tilde{J}$ coordinates:

(16) $$L_{joint}={∥\tilde{J}-J∥}^{2}$$

Therefore, the loss function for this stage of the training process is

(17) $$L_{\mathrm{loss}\;}=\lambda_{\mathrm{pose}\;}L_{\mathrm{pose}\;}+\lambda_{\mathrm{shape}\;}L_{\mathrm{shape}\;}+\lambda_{\mathrm{verts}\;}L_{\mathrm{verts}\;}+\lambda_{\mathrm{joint}\;}L_{\mathrm{joint}\;}$$

where $\lambda_{\mathrm{pose}\;}$ = 5, $\lambda_{\mathrm{shape}\;}$ = 50, $\lambda_{\mathrm{verts}\;}$ = 100, and $\lambda_{\mathrm{joint}\;}$ = 100.

Note that the complete front point cloud in Stage 2 is generated from the depth map rendering of the dataset model, and the number of points in the front point cloud used as input for both training stages is processed to be the same.

## 6. Experimental Results and Analysis

This section first introduces the experimental implementation details, including dataset partitioning, training specifics, and evaluation metrics. Subsequently, ablation studies are conducted on the input point count and point cloud equivariant feature extraction scheme. Finally, qualitative and quantitative comparisons are made with related methods for SMPL model reconstruction to validate the effectiveness of our approach.

### 6.1. Implementation Details

**Dataset**: The dataset was constructed as described in the previous method, with random sampling applied. The final dataset consists of approximately 10,000 data samples. Data from 12 subjects were used for model training, while data from 3 subjects were used for testing. This is the same as the dataset division method used by PTF [33] and IP-Net [13].

**Training**: The model was trained using the Adam optimizer with a learning rate of 0.0001, a batch size of 2, and default settings for other parameters ($\beta_{1}=0.9$, $\beta_{2}=0.999$, $\epsilon =10^{-8}$). First, the back point cloud prediction network was trained independently for 500 epochs. Then, the entire network was trained end-to-end for 25 epochs until convergence. All training procedures were conducted on a server equipped with an NVIDIA Tesla A100 GPU with 40 GB of memory, taking approximately 2 days to complete.

**Evaluation**: The focus of this study is on the robustness and accuracy of SMPL shape and pose estimation from front point cloud. Consequently, two classic metrics were employed: vertex-to-vertex error and joint position error, both measured in mm. For the estimation of body part labels, the prediction accuracy is represented by the percentage of correctly classified labels. The evaluation experiments are conducted on a graphics workstation equipped with a 12th Gen Intel(R) Core(TM) i9-12900K processor, 64 GB of RAM, and an NVIDIA GeForce RTX 3060 graphics card.

### 6.2. Effectiveness of Body Point Cloud Completion

Although we do not concatenate the predicted back point cloud with the front point cloud for point cloud equivariant feature extraction, the back point cloud prediction module can be regarded as a kind of point cloud completion network to a certain extent. Here, we evaluate its effectiveness in body point cloud completion compared to mainstream point cloud completion networks (PoinTr [25], AdaPoinTr [26]). To train PoinTr and AdaPoinTr, we randomly rendered eight different front point clouds with different perspectives within the range of horizontal angle from −10 to +10° and elevation angle from −10 to +10° for the minimum clothing SMPL mesh in the CAPE dataset as missing point clouds. At the same time, we randomly sampled 16,384 points on the minimum clothing SMPL mesh as complete point clouds, and processed the dataset into PCN [34] format for training. The division of the dataset is the same as the methodology of this paper, and the network parameters and training parameters remain the same as in the original paper.

**Figure 8:**
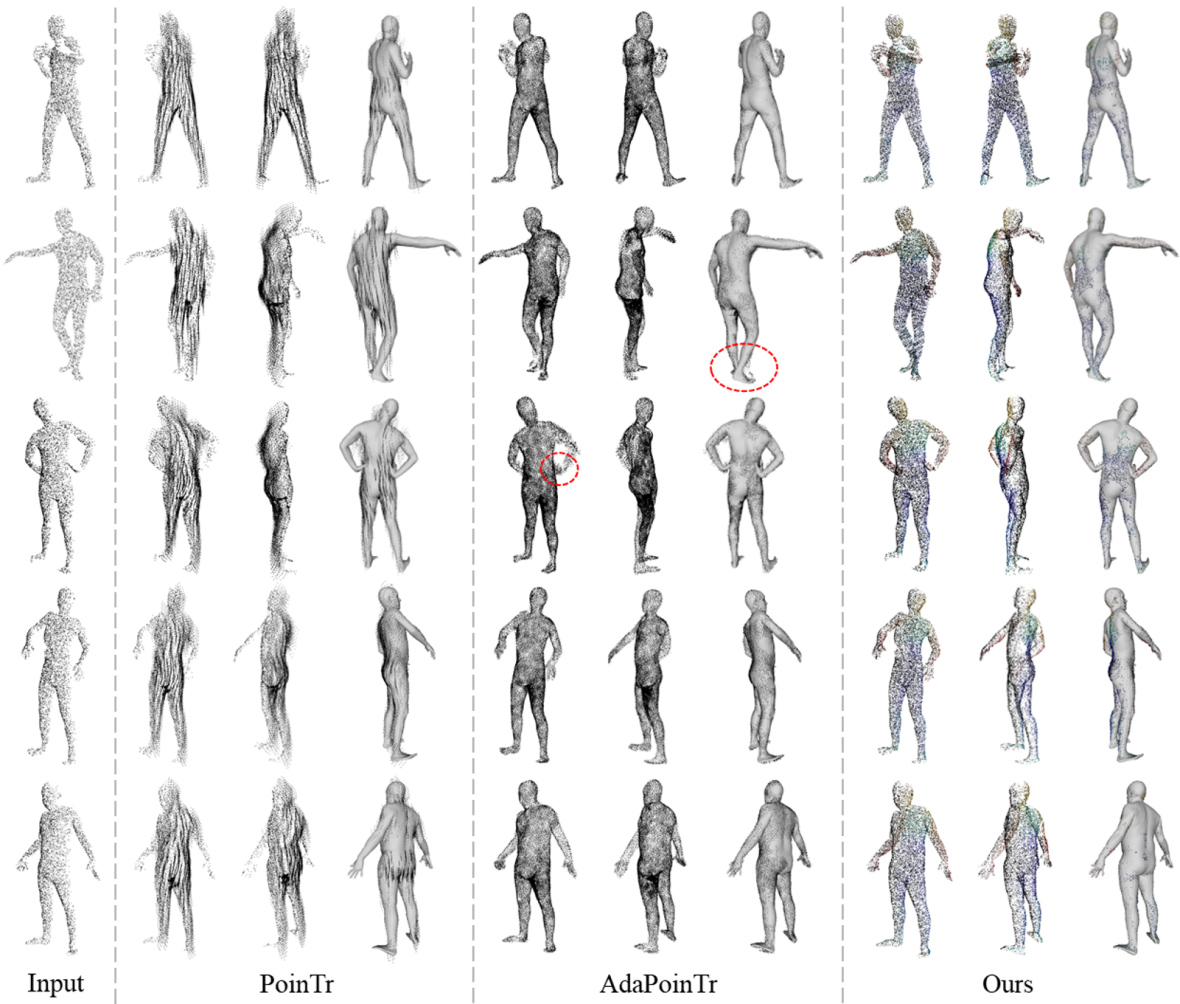
Comparison of relevant point cloud completion methods. The dotted box indicates “ghosting” phenomenon.

The evaluation utilizes common point cloud completion evaluation metrics, namely Chamfer Distance (CD) and Earth Mover’s Distance (EMD), to quantify the accuracy of the completion results, in mm. The performance comparison of different methods is shown in Table 1. Additionally, Figure 8 visualizes the completion performance of different point cloud completion models and verifies the effectiveness of the completion results through overlap with the ground truth model. From the figure, it can be observed that PoinTr and AdaPoinTr focus more on the overall shape of the point cloud, resulting in poor completion performance in the limb regions. For example, PoinTr’s point cloud complementation in the arm region ultimately fails. Although the AdaPoinTr has a significant improvement over the PoinTr complementation, the overlapping view with the ground truth model shows that the shape of the complemented point cloud is not accurate. Furthermore, the distribution of the generated point cloud is not uniform, e.g., more points are concentrated in the crotch. Additionally, when different body parts are close to each other, the ”ghosting” phenomenon (as indicated by the dashed box in Figure 8) is prone to occur. More importantly, these mainstream point cloud completion models inevitably generate a large amount of noise, which seriously affects the point cloud equivariant feature extraction.

In comparison, our model not only achieves better results in terms of CD, EMD, network parameter quantity, and inference time, but also demonstrates a reconstruction effect that is closer to the ground truth shape in qualitative analysis, with uniform point cloud density. In fact, these point cloud completion networks typically use chamfer distance as the loss function. However, the definition of chamfer distance describes the similarity between two sets of point clouds, which fails to truly understand the complex articulated structure of the human body, especially when different body parts are close to each other. Our method completes the body point cloud based on the same parts and corresponding point offsets, with the loss function being the MSE loss between points, which greatly reduces the ambiguity of body point cloud completion. Although the back point cloud prediction module has certain holes in handling self-occlusion in part-based point cloud completion, its prediction of back point clouds is clear, which is crucial for subsequent point cloud equivariant feature extraction.

### 6.3. Ablation Experiment on Point Cloud Equivariant Feature Extraction Scheme

**Figure 9:**
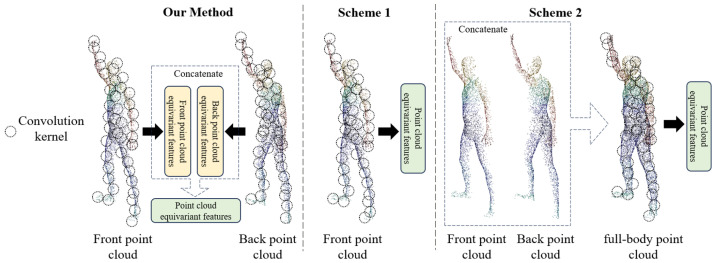
Schematic diagram of different point cloud equivariant feature extraction schemes.

To verify the effectiveness of our FPCR-Net prediction of back point clouds and the extraction of equivariant features from front and back point clouds for SMPL parameter regression, this section designs two additional network architecture schemes, as shown in Figure 9. Scheme 1: Instead of predicting the back point cloud, directly extract equivariant features from the front point cloud. Scheme 2: Concatenate the predicted back point cloud with the front point cloud, and then extract full-body equivariant features through an equivariant point network for use in subsequent modules. All schemes are trained using the dataset in this paper for the same number of iterations.

**Figure 10:**
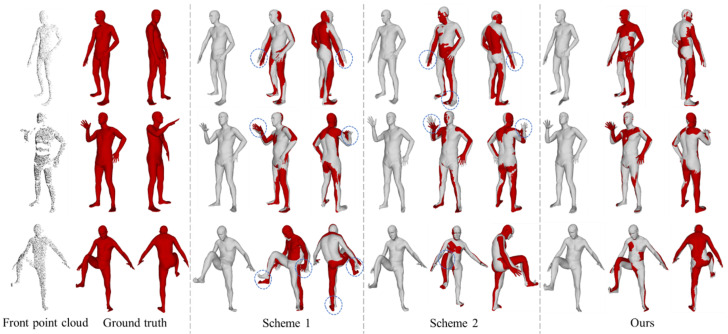
Comparison of reconstructed SMPL models with different point cloud equivariant feature extraction schemes. Dashed boxes indicate large postural deviations.

The qualitative comparison of different point cloud equivariant feature extraction schemes on SMPL model reconstruction is shown in Figure 10. There are significant deviations in the angles of joints between the reconstructed SMPL model and the ground truth in Scheme 1 and Scheme 2, as indicated by the dashed circles in Figure 10. In contrast, our method has a higher degree of overlap with the ground truth. In terms of quantitative comparison, the network parameter quantity and reconstruction accuracy of each scheme are shown in Table 2. Our method achieves the lowest error in both vertex error and joint position error metrics.

Our method introduces a back point cloud prediction module and extracts equivariant features from both the front and predicted back point clouds, thereby capturing body pose and shape information more comprehensively and robustly. Although the model parameter quantity has increased compared to the baseline method, experimental results show that this method reduces vertex error and joint position error by about 37% and 18%, respectively, compared to Scheme 1, and by about 24% and 29%, respectively, compared to Scheme 2. At the same time, the reconstruction time remains within an acceptable range. This fully demonstrates that, despite the increased computational cost, the additional information obtained through predicting back point cloud and extracting equivariant features separately contributes significantly to improving the accuracy and robustness of SMPL model reconstruction.

### 6.4. SMPL Body Shape and Pose Prediction

At present, ArtEq [11] is the only work that directly regresses SMPL parameters from point cloud. However, this method is designed for full-body point cloud regression task. To adapt it to scenarios with only front point cloud input, we retrain it using the dataset in this paper according to the original paper’s method until convergence. Additionally, considering that most contemporary depth cameras are capable of capturing color images, we also compare our method with PostureHMR [18], which regresses SMPL parameters from a single RGB image. The network structure and parameter settings from the original paper are used. The reconstructed models are scaled to match the dimensions of the ground truth models and then aligned using the ICP algorithm.

**Figure 11:**
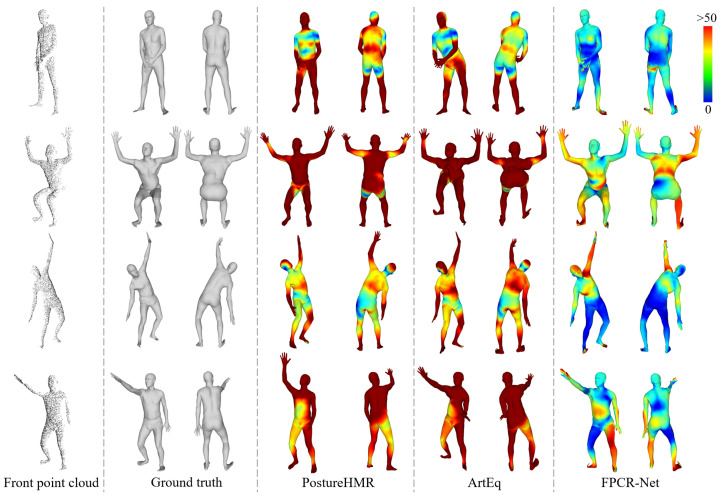
Qualitative comparison of relevant regression methods to reconstruct SMPL models. The vertex error is given by the color map.

The qualitative comparison of the relevant methods for reconstructing the SMPL model is shown in Figure 11. It can be observed that the PostureHMR suffers from significant errors in reconstructing joint rotation angles of the SMPL model compared to the ground truth, due to inherent ambiguities in the 2D to 3D conversion. Although the ArtEq regresses SMPL parameters from the front point cloud, its pose regression is inaccurate, especially when the parts are close to each other; the reconstruction results have a large deviation, which may even lead to reconstruction failure. In contrast, our FPCR-Net can reconstruct the SMPL model more accurately and exhibits higher robustness. Furthermore, even in cases where parts of the front point cloud are missing (such as the almost complete disappearance of the left hand in the last example in Figure 11), FPCR-Net is still able to generate plausible results. Table 3 provides quantitative comparison results of various regression methods for reconstructing the SMPL model. Although all methods can quickly complete the reconstruction of the SMPL model, FPCR-Net achieves the lowest vertex error and joint position error, and its reconstruction accuracy is approximately doubled compared to the ArtEq method, further validating its effectiveness.

**Figure 12:**
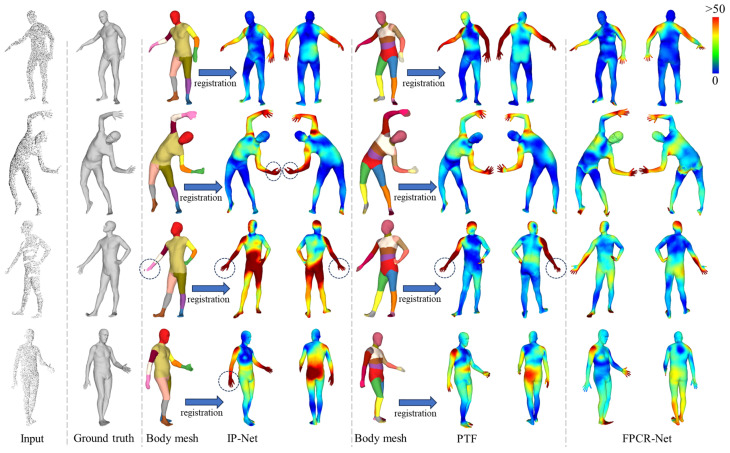
Qualitative comparison with SMPL reconstruction method based on implicit representation. The vertex error is given by the color map. Dashed box significant errors in limb length or rotation angles within the SMPL fitting model.

To comprehensively evaluate the performance of the proposed method, we conducted additional comparative analyses with implicit representation methods, namely IP-Net [22] and PTF [21]. Both IP-Net and PTF process input front point cloud to first predict the complete body mesh, followed by a fitting procedure with a parametric model to achieve reconstruction. Figure 12 illustrates a qualitative comparison of our method and implicit representation-based methods in SMPL model reconstruction. It can be observed that while implicit representation-based methods are powerful, they tend to encounter issues such as broken or rough meshes in detailed regions like the hands, which result in significant errors in limb length or rotation angles within the SMPL fitting model (as highlighted by dashed circles in Figure 12). Furthermore, the quantitative indicators of each method in SMPL model reconstruction are compared in Table 4. The results demonstrate that FPCR-Net achieves vertex and joint position error metrics comparable to those of IP-Net. However, it is worth noting that FPCR-Net is entirely regression-based and does not rely on complex optimization-based fitting processes, thus offering a significant advantage in reconstruction time.

## 7. Limitations and Discussions

Compared with the complete geometric information provided by the full-body point cloud, the single-view point cloud input has inherent limitations in the task of 3D body reconstruction. When the body is in a complex pose, single-view scanning often produces serious self-occlusion. These missing points not only hinder the model’s perception of local details of the body, but also lead to wrong aggregation in the process of feature extraction, which reduces the accuracy of reconstruction, and may even lead to reconstruction failure.

**Figure 13:**
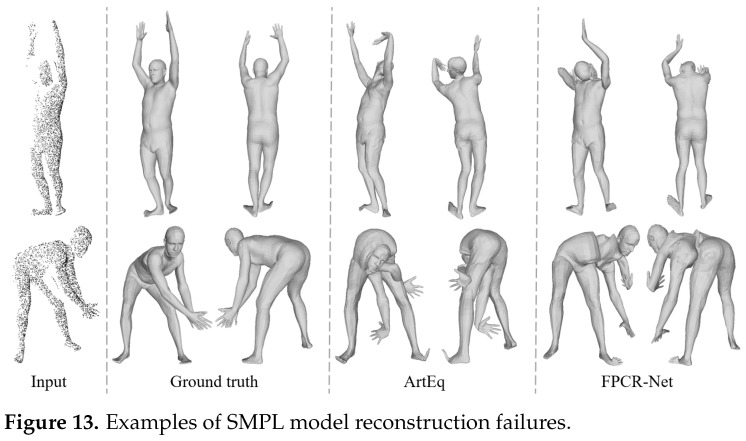
Examples of SMPL model reconstruction failures.

As illustrated in Figure 13, the examples of partial regression failures highlight the limitations of single-view point cloud reconstruction methods in specific scenarios. In the first row of Figure 13, severe self-occlusion in the front scanned point cloud results in significant missing data for multiple key body regions, such as the arm and shoulder. In addition, when multiple body parts are gathered together, as shown in the second line of Figure 13, it will also cause interference to the network body feature extraction. These issues are also inherent limitations of the Arteq method, reflecting common shortcomings of single-view point cloud-based body reconstruction approaches. Nevertheless, in practical applications, such methods prioritize the rapid acquisition of a user’s 3D body model, rather than fine reconstruction under complex poses or extreme perspectives.

## 8. Conclusions

This paper presents FPCR-Net, a novel SMPL parameter regression network based on front body point clouds. Unlike traditional methods that require parametric model registration, FPCR-Net directly predicts SMPL pose and body shape parameters from a front point cloud. The network architecture consists of four key modules: the back point cloud prediction module, the equivariant feature extraction module, the pose prediction module, and the shape prediction regression module. Notably, by predicting the back point cloud and extracting SO(3) equivariant features from both front and back point clouds, FPCR-Net effectively mitigates the rotational ambiguity interference found in full-body point clouds and alleviates the challenges of feature extraction when non-articulated parts approach each other. Experimental results show that the back point cloud prediction module in FPCR-Net outperforms existing methods in terms of point cloud completion accuracy and clarity. Compared to other SMPL parameter regression methods, FPCR-Net significantly improves the accuracy and robustness of vertex and joint position predictions. Furthermore, FPCR-Net’s reconstruction accuracy is comparable to methods based on implicit representations, while its reconstruction efficiency is increased by approximately two orders of magnitude. FPCR-Net is both efficient and accurate, offering broad application potential in areas such as pose estimation, virtual reality, and human–computer interaction, and is especially well suited for rapid body model reconstruction from a single-depth camera point cloud.

## Figures and Tables

**Table 1:** Comparison of human point cloud completion in different networks. Best results are in bold.

Method	CD	EMD	Parameter	Time
PoinTr [25]	402.2	30.4	40.5 M	3.3 s
AdaPoinTr [26]	231.7	22.4	31.0 M	2.5 s
Ours	**187.8**	**17.2**	**7.86 M**	**0.9 s**

**Table 2:** Results for different network architecture design schemes. Best results are in bold.

Design Schemes	Parameter	Vertex Error	Joint Position Error	Time
Scheme 1	**2.0 M**	60.2	49.3	**2.2 s**
Scheme 2	10.3 M	49.7	57.0	3.0 s
Ours	10.6 M	**37.8**	**40.2**	3.2 s

**Table 3:** Comparison of SMPL regression performance of related methods. Best results are in bold.

Method	Input	Parameters	Vertex	Joint Position	Time
PostureHMR [18]	Front RGB image	29.4 M	71.4	82.7	**1.2 s**
ArtEq [11]	Front point cloud	**0.9 M**	66.5	73.1	2.3 s
FPCR-Net	Front point cloud	10.6 M	**37.8**	**40.2**	3.2 s

**Table 4:** Quantitative comparison with SMPL reconstruction method based on implicit representation. Best results are in bold.

Method	Parameters	Vertex	Joint Position	Time
IP-Net [22]	35.0 M	35.3	42.6	311.4 s
PTF [21]	34.1 M	**24.1**	**31.4**	298.3 s
FPCR-Net	**10.6 M**	38.1	42.2	**3.2 s**

## Data Availability

Dataset available on request from the authors.
